# What’s the best surgical treatment for patients with cervical radiculopathy due to single-level degenerative disease? A randomized controlled trial

**DOI:** 10.1371/journal.pone.0183603

**Published:** 2017-08-29

**Authors:** Roland D. Donk, André L. M. Verbeek, Wim I. M. Verhagen, Hans Groenewoud, Allard J. F. Hosman, Ronald H. M. A. Bartels

**Affiliations:** 1 Via Sana Clinics, Department of Orthopedic Surgery, Mill, the Netherlands; 2 Radboud university medical center, Department for Health Evidence, Nijmegen, the Netherlands; 3 Canisius Wilhelmina Hospital, Department of Neurology, Nijmegen, the Netherlands; 4 Radboud university medical center, Department of Orthopedic Surgery, Nijmegen, the Netherlands; 5 Radboud university medical center, Department of Neurosurgery, Nijmegen, the Netherlands; 6 Canisius Wilhelmina Hospital, Department of Neurosurgery, Nijmegen, the Netherlands; UNITED STATES

## Abstract

**Background:**

To investigate the efficacy of adding supplemental fusion or arthroplasty after cervical anterior discectomy for symptomatic mono-level cervical degenerative disease (radiculopathy), which has not been substantiated in controlled trials until now.

**Methods:**

A randomized controlled trial is reported with 9 years follow up comparing anterior cervical anterior discectomy without fusion, with fusion by cage standalone, or with disc prosthesis. Patients suffering from symptomatic cervical disk degeneration at one level referred to spinal sections of department of neurosurgery or orthopedic surgery of a large general hospital with educational facilities were eligible. Neck Disability Index (NDI), McGill Pain Questionnaire Dutch language version (MPQ-DLV), physical-component summary (PCS), and mental-component summary (MCS) of the 36-item Short-Form Health Survey (SF-36), and re operation rate were evaluated.

**Findings:**

142 patients between 18 and 55 years were allocated. The median follow-up was 8.9±1.9 years (5.6 to 12.2 years). The response rate at last follow-up was 98.5%. NDI at the last follow-up did not differ between the three treatment groups, nor did the secondary outcomes as MPQ-DLV and PCS or MCS from SF-36. The major improvement occurred within the first 6 weeks after surgery. Afterward, it remained stable. Eleven patients underwent surgery for recurrent symptoms and signs due to nerve root compression at the index or adjacent level.

**Conclusions:**

This randomized trial could not detect a difference between three surgical modalities for treating a single-level degenerative disk disease. Anterior cervical discectomy without implant seems to be similar to anterior cervical discectomy with fusion by cage stand-alone or with disk prosthesis. Due to the small study sample size, this statement should be considered as inconclusive so far.

**Trial registration:**

ISRCTN41681847

## Introduction

Symptomatic degeneration of a cervical intervertebral disk is encountered frequently in daily practice with irradiating pain in the arm with or with loss of sensibility or motor function as clinical presentation. The incidence varies between 0.83 and 1.79 per 1000 person-years[1, 2]. In most instances, the disk will recover spontaneously without surgical intervention[3]. In case of severe pain or pain not responding to conservative treatment, surgery is a valid and effective option[4].

The anterior approach is the most often used of the surgical options. In the 1950s and 1960s, cervical anterior discectomy without (ACD) and with fusion (ACDF) were developed and propagated. Although sound evidence is still lacking for the superiority of ACDF, it serves as gold standard. Despite the high rate of recovery of non-operative therapy, an almost eight-fold increase in utilization of ACDF from 1990 to 2004 was recently reported.[5] Currently, plate fixation is considered standard for ACDF. Other fusion methods are the use of only a bone graft or a cage stand-alone. In the past two decades, another implant gained popularity, the disk prosthesis. In literature arthroplasty (ACDA) is now compared with ACDF by plate fixation. However, there has never been any definitive conclusion to the discussion of the superiority of ACDF; therefore, it is of utmost importance to complete this discussion, since more complications due to hardware failure may be involved, and the costs are significantly higher. ACDA has not been compared with ACDF with cage stand-alone, whereas the dissection for the latter is nearly the same as for ACDA, which may cause similar perioperative complication rates.

Despite statistically significant superiority, the clinical outcome after ACDA and ACDF with plate fixation is similar regarding clinical relevance.[6, 7] At present, research is focused mainly on degeneration of the adjacent intervertebral disk,[8] which is diagnosed radiologically. However, this is a surrogate outcome, and its clinical importance is unknown. The causative relation to surgery is also subject of debate.

Since implants are costly, the discussion should start with the question whether implants are needed in case of surgical therapy for single-level disease. This is the first study that investigates whether the patient-reported clinical outcome differed between patients who were treated by either ACD, ACDF with cage stand-alone, or ACDA.

## Methods

### Study design and oversight

The guidelines of the CONSORT 2010 statement were followed (S1 File)[9].

Registration of the trial in the registry was done shortly after the starting recruitment of patients, since the authors (at that time) were not aware of the fact that registration in an international register was also necessary since it was already registered in a national register. The authors confirm that all ongoing and related trials for this drug/intervention are registered. Patients were enrolled between October 5^th^, 2003 and June 10^th^ 2010 in a single center (the Canisius Wilhelmina Hospital, Department of Neurosurgery, Nijmegen, the Netherlands) in a randomized controlled trial. The protocol has been described earlier (S2 and S3 Files.[10]

Patients were eligible if they suffered from a radicular syndrome in the arm due to one-level cervical degenerative disease of an intervertebral disk at MRI and the involved level was still mobile at dynamic radiographs. They were assigned to surgical treatment consisting of cervical anterior discectomy followed by one of the following three surgical options: fusion by cage stand-alone (ACDF), arthroplasty (ACDA), or no implant at all (ACD). After written informed consent, patients were randomized using a closed-envelope system delivered by an independent co-worker of the medical administrative subdivision of the department.

The trial design was a prospective, double blind, single center randomized study with a three arm parallel group design. The experimental group was ACDA, whereas ACDF and ACD were control group. In the final analysis, it was considered as a superiority design. The type of randomization was 1:1:1. The evening before surgery, the treating surgeon was informed to which group the patient had been allocated.

Although designed as a multiple centre study, the commitments from other centers to contribute were not fulfilled due to several reasons. Reasons were the introduction of more promising and less technically demanding implants on the market, and the lack of financial support.

Although a formal interim analysis was not planned nor made, because it was expected that adding a different implant to a very common procedure would not result in a dramatic positive or negative effect that would justify terminating the trial, whereas the sample size would increase, the inclusion ended before reaching the predefined sample size. After the publication of a meta-analysis indicating that a clinical difference was not present comparing cervical anterior discectomy with fusion and with arthroplasty.[11], we could not justify the continuation of the trial, because the costs for the disk prosthesis were five times higher than a cage stand-alone.

The primary outcome measure was the Neck Disability Index (NDI) at five years. Due to the longer inclusion time and our interest in the log term results, we decided after internal decision to send all patients questionnaires about the NDI in order to have a better impression of the effect of surgery after longer follow-up for the primary outcome measurement. This was done at the moment the last patient completed the five year follow-up. All patients were contacted again and asked to complete the NDI. The last follow-up for all patients was December 1^st^, 2015.

Secondary outcome measurements were McGill Pain Questionnaire Dutch language version (MPQ-DLV), numeric rating scale (NRS) arm and NRS neck, physical-component summary (PCS) and mental-component summary (MCS) of the 36-item Short-Form Health Survey (SF-36), complications, and reoperations.

Postoperative data collection started 6 weeks postoperatively, and patients were followed for 5 years. At the last follow-up, patients were also asked to complete NRS arm and NRS neck questionnaires estimating the pain during the previous 24 hours.

### Patients

All adult patients aged between 18 and 55 years with monoradicular signs and/or symptoms in the arm due to a herniated cervical intervertebral disk and/or an osteophyte at MRI without a history of any cervical spine surgery were eligible. In the original protocol a maximum age was written of 50. We assume that this was a miswriting since in the trial registration the maximum age of was 55. In the subsequent publication[10] the maximum age was also set at 55. The radiological findings should be in accordance with the clinical presentation, and the involved level should be mobile at dynamic radiographs of the cervical spine.

Patients were screened for eligibility after referral. The surgeon offered the possibility of participation to the trial. After at least 48 hours, the patients were contacted again and asked for informed consent.

### Patient involvement

We did not involve patients or lay people in the design of the study, since the basic cervical anterior discectomy was already a well-known and accepted treatment. We were interested if adding an implant would be of benefit for the patient. For these purposes we have chosen outcome measures that were known to reflect the clinical situation and daily burden of a patient regarding disability, pain and quality of life. To investigate the benefit we were interested in outcome measurements reported by the patients themselves. Before the study, we extensively studied the burden of participating to the trial for the patients since it was also part of the approval procedure for the ethical committee.

During the study, however, the method for follow-up has significantly been changed since patients requested not to be obliged to visit the outpatient clinic in order to complete the scheduled follow-up moments if they had no complaints. They were willing to complete the questionnaires at home and return them by flat mail.

### Interventions

All patients underwent a standard anterior cervical discectomy with bilateral decompression of the nerve roots. If the patients were allocated to ACD, the wound was closed; if they were allocated to ACDF, a cage stand-alone filled with autologous bone was implanted (Brantigan cervical I/F cage, DePuy Spine, Inc., Raynham, MA, USA), and in case of ACDA, a Bryan disk prosthesis (Bryan disk prosthesis, Medtronic, Memphis, TN, USA) was implanted according to the guidelines provided by the company. Postoperatively, none of the patients was prescribed a collar. To prevent heterotopic ossification, only the patients allocated to ACDA were prescribed meloxicam for 2 weeks.

All (four) trial surgeons were senior spinal surgeons experienced in the three types of intervention. Institutional review board approval was obtained (The Ethics Committee CMO Arnhem-Nijmegen, CMO-nr: 2002/200; date of approval May 14, 2003). There was no industry funding.

### Outcome measures

The primary outcome was a change in NDI score (scale 0 to 50 points) at the last follow up in November 2015. The NDI is a well known and validated (in multiple languages) outcome-measurement instrument to assess self-rated disability in patients with neck pain.[12–14]

Secondary outcomes were the MPQ-DLV, SF-36, complications, re-operations, and visits to physicians or therapists concerning neck problems after the index surgery for advice or conservative treatment.

MPQ-DLV is a questionnaire that includes several domains. At the moment of completion, the MPQ-DLV and the visual analog scale (VAS) should be rated; whether the complaints were minimal or maximal should also be indicated. A description of the pain should also be given, chosen from a list of adjectives. The number of adjectives was counted (number of word chosen-total [NWC-T]), as well as the sum of the ranks belonging to each adjective (pain rating index total [PRI-T]).

Patients were encouraged to complete the SF-36, MPQ-DLV, and NDI questionnaires themselves, or with the assistance of an independent physician assistant, before they visited or contacted their physician. Patients who wanted to participate, but did not want to visit the outpatient clinic, were offered the possibility to complete the questionnaires at home and return them as hard copy by mail. Baseline NDI, SF-36, and MPQ-DLV were derived carried through 60 months. NDI was also derived at the last follow-up, as was the NRS arm and NRS neck. The response rate was expected to decrease at every follow-up visit during the first 2 years, a well-known phenomenon.[15–17]

For the final follow-up, we emphasized the importance of completing the questionnaires and reminded the patients to do so, if they had not responded.

To optimize participation further, we focused at the last follow-up on the primary outcome (NDI) and the NRS arm and NRS neck. We did not use the MPQ-DLV since it had been shown that responsiveness was higher for NRS compared with VAS,[18] which is only a part of the MPQ-DLV. A high correlation has been found between VAS and NRS supporting interchangeable application.[19]

Recently, a good outcome was also defined as NDI ≤7.[20] Apart from comparison of the NDI value among groups, the proportion of good outcome was also evaluated, although it was not included in the original protocol.

Individual data can be assessed in the supplementary S4 File as well as the outcome measured by NDI in S5 File.

### Statistical analysis

We changed the noninferiority assumption into a superiority assumption. We justified this change by the fact that we assumed originally that the three methods resulted in at least similar clinical results, and we expected better results for ACDA.

In the original protocol a 20% difference in excellent outcome was the base for the sample size calculation. An excellent outcome, however, was not exactly defined. Since NDI was a primary outcome measurement, the statement about difference in excellent outcome was interpreted as a difference in NDI.

The total sample size using NDI measuring on a numeric scale (0/50) should be a minimum of 243 patients in order to detect a difference of 10 (or 20% if the percentage scale was used ranging from 0 to 100%). The alternative hypothesis considered a difference of 10 points or 20% in the NDI as clinically relevant. While designing this study, information regarding the minimal clinically important difference was lacking, but recent studies confirmed our assumption.[21, 22] We estimated a dropout rate of 10% and therefore estimated that the trial needed to include 270 patients. A dropout rate of 10% was chosen since it was assumed that a certain amount of people would not participate in the trial after inclusion. Since this number varied comparing several trials we arbitrarily have chosen for 10% in order to have the greatest change to perform an analysis on the previously calculated sample size.

For analysis the intention to treat principle was followed. Analyses of the primary outcome were performed including all patients that had completed the questionnaires at their last follow-up. From the two patients that were lost to follow-up, the baseline data were included, as well as the data at their last follow-up moment. Missing data were not imputed. Only the available data were analysed. Stratification was not applied.

For NDI as the primary outcome measure, analyses were done using a linear mixed fixed effects model with variance components as covariance type and only a random intercept. In this model treatment group (factor), moment of measurement (factor), and baseline score (covariate) were used to explain the dependent variable (NDI). Age (covariate), enrolment time (covariate), surgeon (factor), and gender (factor) were incorporated in the model, in order to correct for possible confounding.

The proportion of patients with a good outcome within each treatment group was also compared. The same technique with similar variables was used for analyses of MPQ-DLV and SF-36 for two years postoperatively and at 5 years. Analyses for NRS arm and neck were set at the last follow up (December 2015).

The NRS arm and NRS neck at the last follow-up between groups were analysed separately and investigated using the one-way ANOVA method. Two analyses were done: (1) including all patients irrespectively of the intervening treatment simulating a real life situation, and (2) excluding those patients that underwent additional surgery. HG and RB analysed the data.

Baseline characteristics were compared between groups using chi-square tests or for categorical data, and one-way ANOVA techniques for continuous data. Numeric data are represented by mean value ± standard deviation (SD). Results of the analyses by the mixed model are represented as mean, standard error and 95% confidence interval (95% CI). For the statistical analyses, SAS version 9.2 (SAS Institute Inc. Cary NC, USA) was used.

## Results

### Patients

Overall, 272 patients were eligible for inclusion in the trial after screening. However, 18 patients explicitly had a clear preference for one treatment, and 112 refused to participate. Finally, 142 patients gave informed consent and were randomized. The mean age of the study population was 44.9±6.5 years; 50% were female. Baseline characteristics are presented in Table 1, and the distribution of the operated level is presented in Table 2. For 140 (98.6%) patients, the median last follow-up was 8.9±1.9 years (range 5.3 to 12.2 years).

**Table 1:** Baseline characteristics of included patients allocated to anterior cervical discectomy without any implant (ACD), anterior cervical discectomy with fusion by cage stand-alone (ACDF), or anterior cervical discectomy with arthroplasty (ACDA) (All characteristics were similar between groups without reaching statistical significance for any difference). Numerical data represented as mean ± SD.

	ACD	ACDF	ACDA
Age—yr	44.3±5.6	43.1±7.5	44.1±6.4
Gender (F/M)	23/22	22/25	26/24
Smoking (Y/N)	16/29	24/23	27/23
Alcohol consumption (Y/N)	20/25	24/23	27/23
NDI	17.1±6.4	18.8±7.4	18.8±7.5
SF-36 PCS	43.6±12.3	44.0±11.0	44.1±13.9
SF-36 MCS	62.1±18.8	55.7±21.1	58.3±22.2
VAS minimum	21.9±19.2	26.9±21.9	30.1±23.8
VAS maximum	71.6±26.6	68.0±29.1	66.4±29.9
VAS moment	41.9±25.4	39.5±26.0	47.6±29.6
NWC-T	10.5±4.7	8.6±4.8	8.1±4.8
PRI-T	18.5±9.3	15.2±10.2	14.7±10.9
Total	45	47	50

**Table 2:** Surgical level in relation to procedure (ACD, ACDF, or ACDA) statistical difference was not reached (P = 0.232).

Level	Total	ACD	ACDF	ACDA
C4C5	3	1	2	0
C5C6	66	26	19	21
C6C7	73	18	26	29
**Total**	142	45	47	50

Fifty patients were allocated to ACDA, 47 to ACDF, and 45 to ACD. No differences regarding baseline characteristics were present between the treatment groups. One patient allocated to ACDA died due to a cause unrelated to the intervention, and one patient allocated to ACDF refused to complete the last questionnaire.

**Fig 1:**
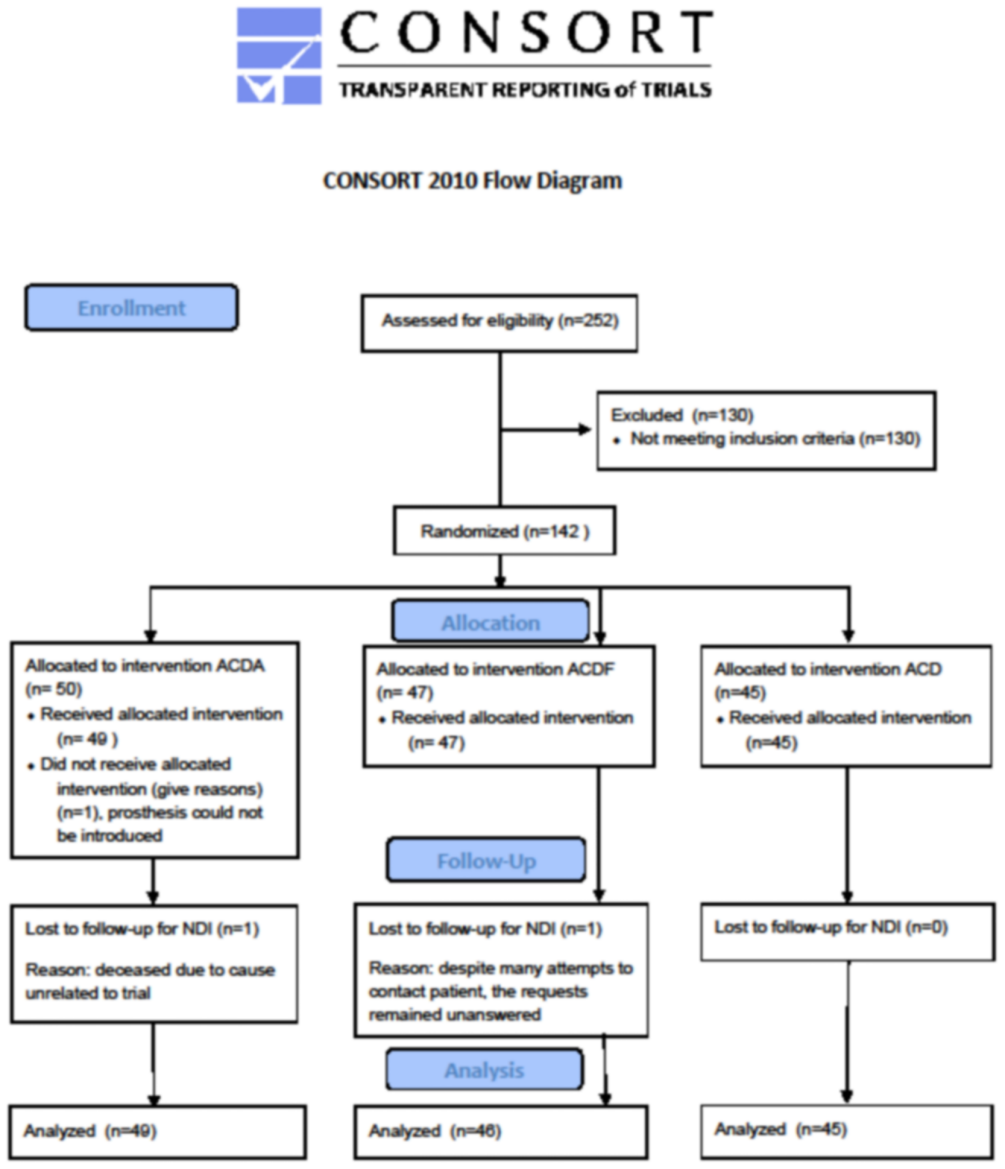
Flow diagram according to Consort.

One patient was allocated to the arthroplasty group, but intraoperatively it was not possible to introduce the disk prosthesis and a cage was implanted instead. According to the intention-to-treat principle, this patient remained within the arthroplasty group for analysis. The flow diagram according to Consort is represented in Fig 1.

### Primary outcome

**Fig 2:**
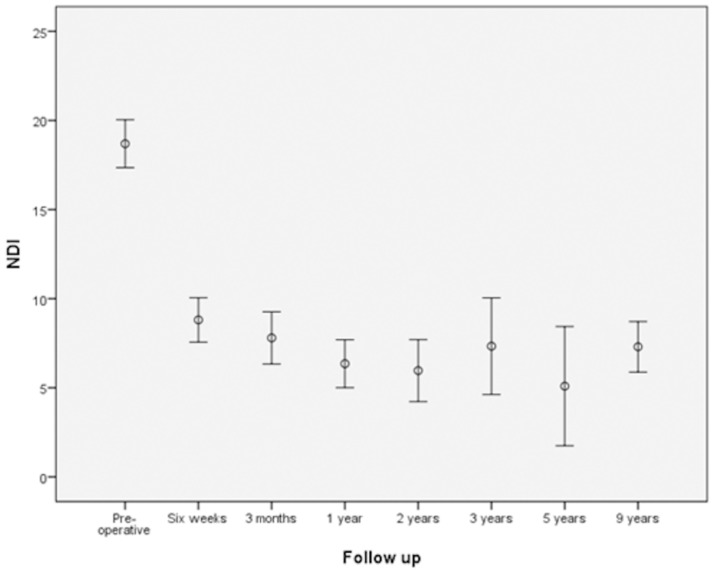
NDI with 95%CI at different follow-up moments for the complete sample.

**Fig 3:**
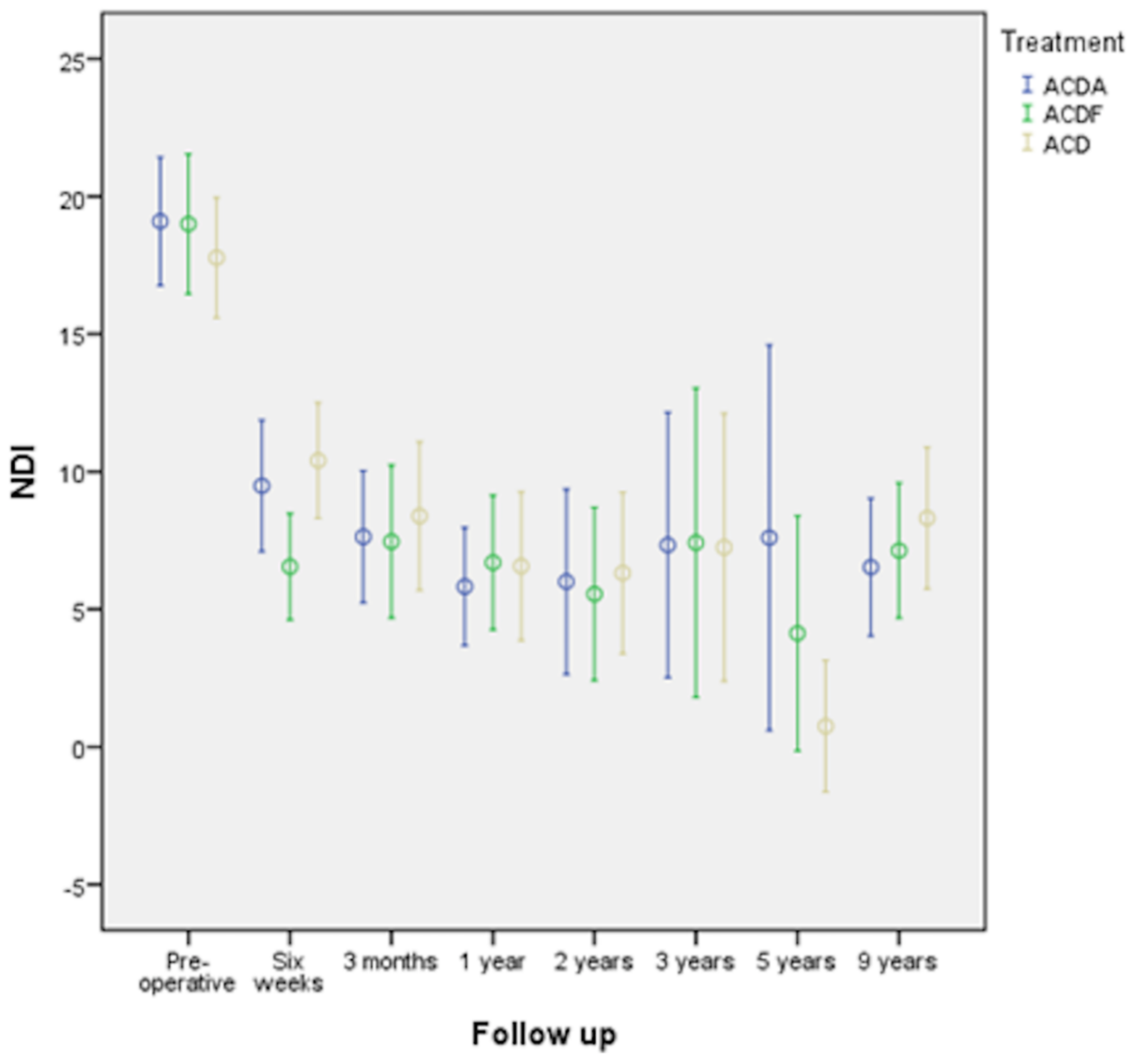
NDI with 95% CI at each follow-up moment and per treatment modality.

The outcome was dependent upon the baseline score (P = 0.009). Gender, surgeon, time to enrolment and age did not affect the outcome between treatment groups. At two years the primary outcome NDI improved 13.4 ± 0.8 points compared to baseline. This difference was statistically significant (P = 0.009). A statistically significant difference between the three groups was absent (Table 3, Figs 2 and 3).

**Table 3:** Estimated marginal mean values of NDI at different follow-up intervals, based on the linear mixed model computed for baseline NDI score of 18.75*.

			95% Confidence Interval		Number of patients		
Postoperative Follow-Up	Mean	Standard Error	Lower Bound	Upper Bound	ACD	ACDF	ACDA
6 weeks	9.2	0.846	7.6	10.9	32	34	36
3 months	7.7	0.846	6.0	9.4	31	36	39
1 year	6.5	0.858	4.9	8.2	30	34	35
2 years	5.5	0.958	3.6	7.4	19	19	24
3 years	7.1	1.046	5.0	9.2	12	13	18
5 years	6.0	1.259	3.5	8.5	4	9	10
9 years	7.5	0.829	5.8	9.1	45	46	49

* At mixed models with fixed effects, only the difference between the preoperative NDI and at 6 weeks’ follow-up reached statistical significance. During the remaining follow-up, it remained stable.

At the last follow-up and compared with baseline NDI improved to 7.5 ± 8.5. A statistically significant difference between the groups was absent (P = 0.324). The only clear and statistically significant improvement in NDI was seen between the measurements pre-operatively and 6 weeks postoperatively. Afterward, a clinically relevant change of NDI did occur anymore during follow-up. The mean difference of NDI between 2 years postoperatively and the last follow up was 2.0 ± 0.7 (p = 0.009). Between the treatment groups a statistically significant difference did not exist (Table 4).

**Table 4:** The difference in estimated marginal means between groups computed with linear mixed model.

Treatment pair		Mean difference	SE	df	P	Lower bound 95%CI	Upper bound 95%CI
ACDA	ACDF	-0.003	1.308	110.793	0.998	-2.595	2.590
ACDA	ACD	-1.659	1.394	108.981	0.237	-4.422	1.104
ACDF	ACD	-1.656	1.429	109.095	0.249	-4.489	1.176

If the patients who underwent a surgical procedure after the index procedure (n = 11) were not incorporated in the analysis, NDI improved to 6.5±7.9, without any statistically significant difference between the treatment modalities (P = 0.832). Of the patients treated by ACDA, 73.5% had a good outcome as defined by a score of NDI ≤7, by ACDF 60.9%, and ACD 57.8% at their last follow-up, but this was not statistically significant (P = 0.239).

The enrolment time to model time[23] did not affect the outcome and was, therefore not included in the model. Gender, age, and surgeon were not related to any clinical relevant outcome measurement.

### Secondary outcomes

**Fig 4:**
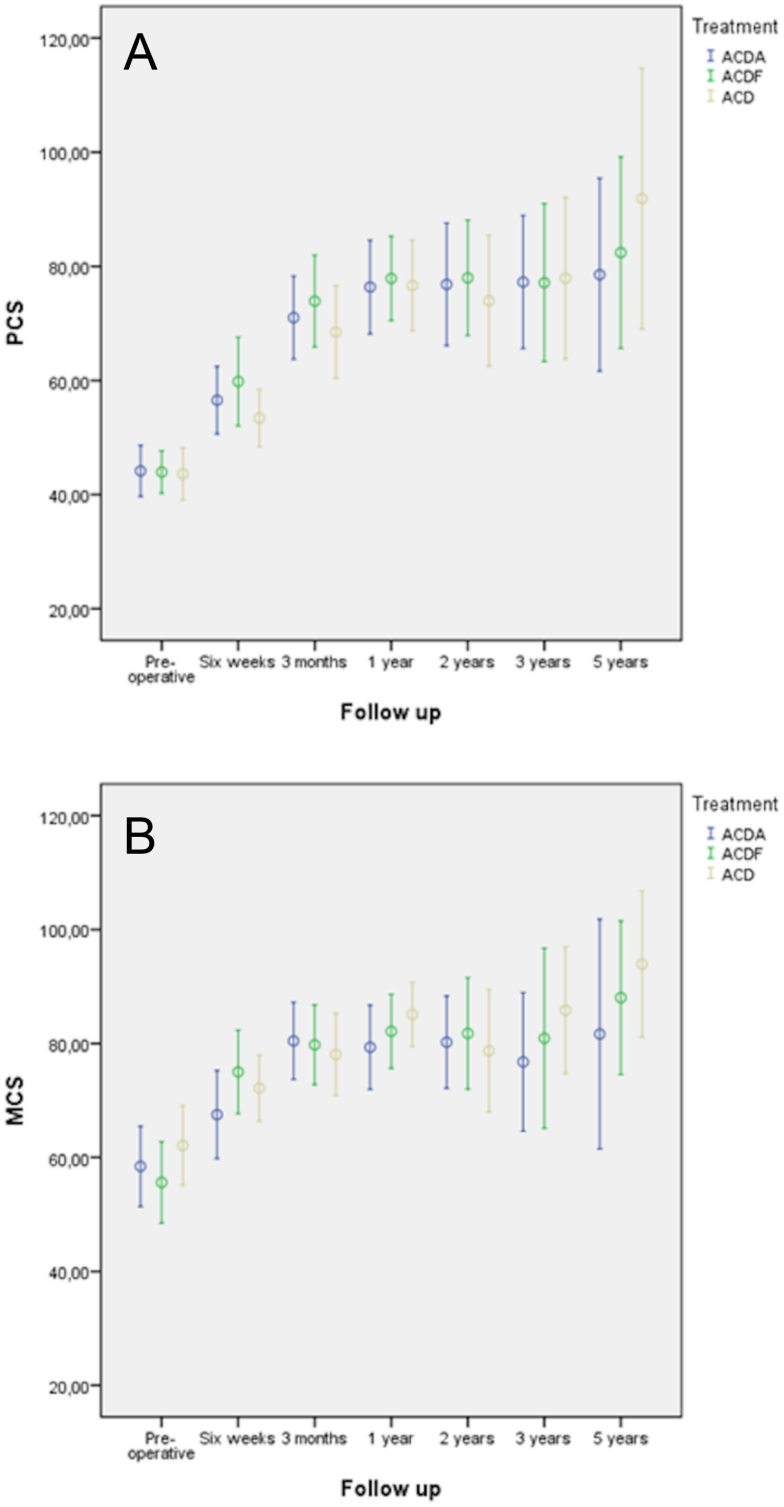
Graph depicting PCS (A) and MCS (B) with 95% CIs at different follow up and for each treatment.

Regarding the summary scales of SF-36 at two years the mean improvement of PCS was 32.1±2.5. A statistical difference between the treatment modalities was not found at any follow-up moment (P = 0.873). MCS improved on average 22.8±2.1 without any statistically significant difference between the groups at any follow-up moment (P = 0.874) (Fig 4).

**Fig 5:**
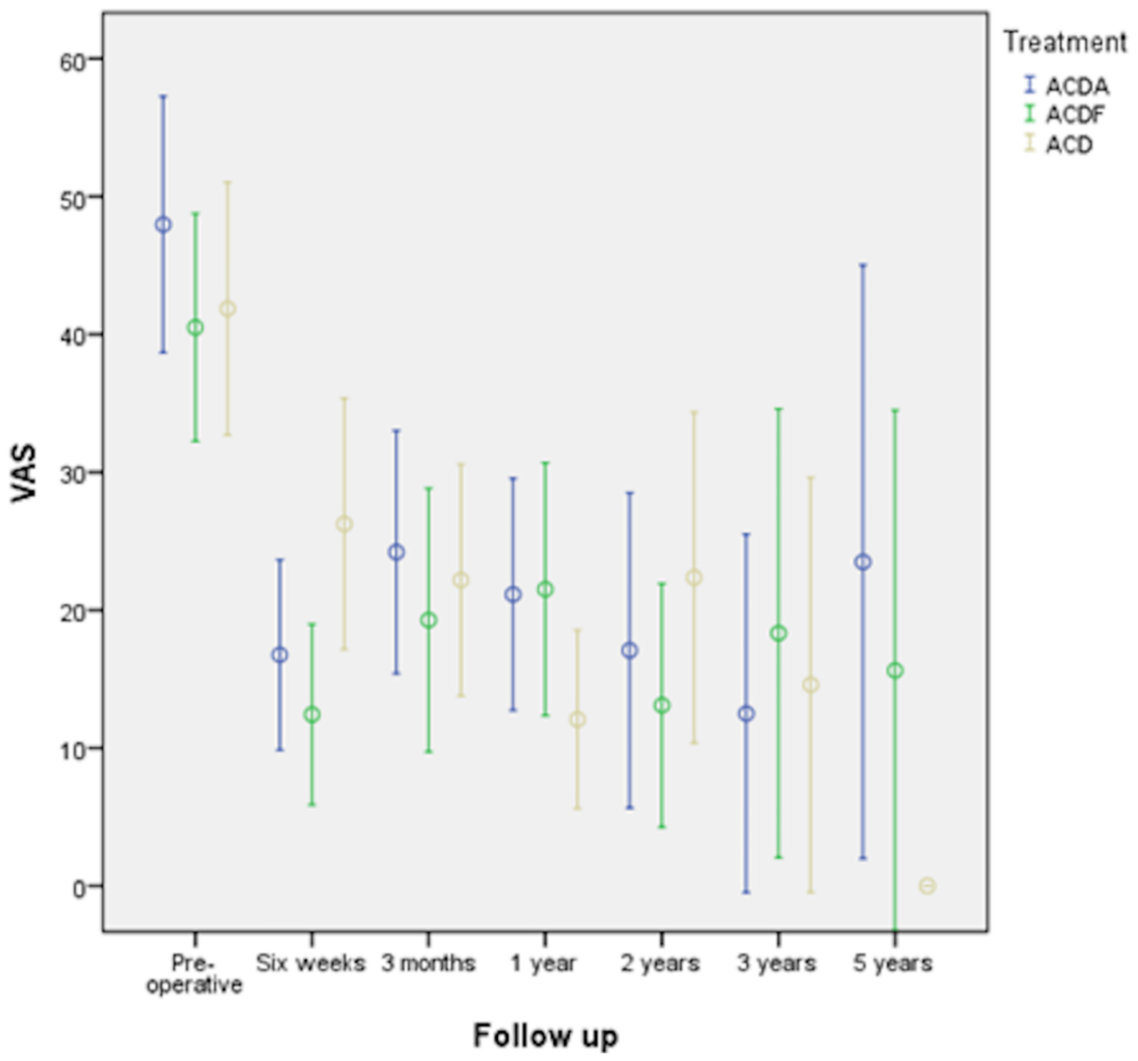
VAS at the moment of completing questionnaire with 95% CI at different follow-up moments until 5 years.

VAS as part of the MPQ-DLV improved to 17.3±24.0, 9.2±16.5 when the complaints were minimal, and 24.4±31.4 if maximal. Only the VAS at the moment of completing the questionnaire is shown in Fig 5. All others had a similar pattern to NWC-T and PRI-T. NWC-T was 4±5, and PRI-T was 6.3±9.7. None of them reached statistical significance between the treatment modalities (VAS at moment completing questionnaire: P = 0.429, VAS minimal pain: P = 0.534; VAS maximal P = 0.593; NWC-T: P = 0.690; PRI-T: P = 0.657).

MPQ-DLV was completed by only 22 patients at 5 years. Therefore, we represent them only in the graphs (Fig 5). Instead, we mention the NRS arm and NRS neck, which were obtained at the last follow–up in 140 patients. NRS arm was 1.8±2.5, and NRS neck was 1.9±2.6. A statistical difference between the treatment groups was not present (P = 0.622 and 0.496, respectively).

### Complications

In 13 patients (9.2%), complications occurred that were not related to signs or symptoms of recurrent compression or nerve root involvement at the adjacent segment (Table 4). Urinary tract infections, pulmonary infections, deep venous thrombosis, pulmonary embolism, or deep wound infections did not occur. A superficial wound infection was present in one (0.7%) patient, hoarseness was reported in four (2.8%) patients, dysphagia in seven (4.9%) patients, and a postoperative hemorrhage warranting surgical re-exploration in one (0.7%) patient (Table 5).

**Table 5:** Complications related to treatment group (Number/Percentage of group).

Complication	ACD	ACDF	ACDA
Number of patients	45	47	50
Urinary tract infection, n (%)	0 (0)	0 (0)	0 (0)
Pulmonary infection, n (%)	0 (0)	0 (0)	0 (0)
Deep venous thrombosis/pulmonary embolism, n (%)	0 (0)	0 (0)	0 (0)
Superficial wound infection, n (%)	0 (0)	1 (2.1)	0 (0)
Deep wound infection, n (%)	0 (0)	0 (0)	0 (0)
Hoarseness, n (%)	3 (6.7)	1 (2.1)	0 (0)
Dysphagia, n (%)	1 (2.2)	4 (8.5)	2 (4.0)
Postoperative hemorrhage, n (%)	1 (2.2)	0 (0)	0 (0)
Total, n (%)	5 (11.1)	6 (12.8)	2 (4.0)

### Recurrent nerve root symptomatology

Eleven (7.8%) patients underwent surgery due to recurrent signs or symptoms related to compression of a nerve root at the index level in three (2.1%) patients and at the adjacent level in eight (5.6%) patients (Table 6). The difference between groups did not reach statistical significance (P = 0.132).

**Table 6:** Surgery for recurrent signs and symptoms due to nerve root compression at the index level or adjacent segment.

Procedure	ACD	ACDF	ACDA
Surgery for adjacent segment disease, n (%)	3 (6.7)	5 (10.6)	0 (0)
Surgery for recurrent compression at index level, n (%)*	1^†^ (2.2)	1 (2.1)	1 (2.0)
Posterior surgery	*1* (*2*.*2)*	*1* (*2*.*1*)	*1*^‡^ (*2*.*0*)
Anterior surgery	*1* (*2*.*2*)	*0(0)*	*0* (*0*)
Total, n	4	6	1

* Approaches for the surgery at the index level is subdivided in anterior or posterior approach.

^†^ One patient was also operated anteriorly because of insufficient result of the first posterior re-exploration.

^‡^ One patient visited the outpatient clinic for recurrent signs and symptoms before completing the NDI questionnaire. This crossed the radiological examinations, after which she was offered surgical therapy for recurrent stenosis at the index level. She was not included in this analysis.

### Consultation of physicians or therapists

Of 140 patients at the last follow-up, 58 (41.4%) consulted at least once a physician or therapist after the index surgery for problems relating to their neck. The number of patients was equally divided among treatment allocations (ACDA 19, ACDF 19, ACD 20) without any statistically significant difference (P = 0.872). The consulted caregivers (with the number of patients who visited the caregiver in parentheses): physiotherapist (26); chiropractor (2); osteopath (1); neurosurgeon (15); orthopedic surgeon (5); general physician (1); pain consultant (6); and neurologist (5). Some patients consulted more than one caregiver. A difference between treatment groups did not exist (P = 0.144).

## Discussion

This study is unique since three surgical options were compared: ACD, ACDF with cage stand-alone, and ACDA. The follow-up period is the longest in literature, and the response rate for primary outcome NDI is very high (98.6%).

For the first time, the clinical outcome of ACDA is compared with ACDF with cage stand-alone or ACD. Until now, arthroplasty has been compared only with ACD with fusion with plate fixation. Comparing ACD with arthroplasty and ACD with fusion by plate is comparing two different surgical methods, since the dissection is wider and slightly different in case of the implant of a cage. ACDF with cage stand-alone will resemble more the technique of ACDA.[24] In the current trial, the only difference is whether an implant is chosen and, if so, which implant.

It is remarkable that ACDF is considered as the gold standard, since sound evidence is still lacking in literature. In one study comparing four groups, a statistically significant difference regarding in favour of an additional implant was found after short-term follow-up.[25] The authors of that study commented on the findings that the results were flawed by the small sample size of 125 patients in total.[25] A recent systematic review did not show any clinical superiority of ACDF.[26]

Patients want a treatment that provides them with long-term relief of signs and symptoms, so focus on clinical outcome is important. Therefore, we did not remove from our analysis the patients who have been operated on after the index surgery. This analysis shows clearly what the result of a treatment is after 9 years, including intervening surgical therapies. We did not focus on radiological outcome. In our opinion, surrogate outcomes such as radiological deterioration of adjacent levels without any clinical sign or symptom are not relevant for patients.

In the end, comparing ACD with ACDF and ACDA, the clinical result is similar. Irrespective of the treatment, there is a small change indicating that an additional surgical procedure is needed. Although not statistically significant, it seems that surgery for adjacent segment disease is less often provided for ACDA. Although proponents of the use of cervical disk prostheses claim that prevention of adjacent segment disease is their major benefit compared with ACDF with plate fixation, meta-analyses still show contradicting results.[27–29]

Although our results will contribute to future meta-analyses on this topic, we do not feel confident to recommend disk prostheses as a standard option. Health economics should be considered. Since clinical outcome is not involved in the end, societal costs and hospital costs are involved. These differ between countries. Furthermore, it is important to calculate the number to treat to prevent one extra patient from developing adjacent segment disease. If the costs of an implant are relatively very high (as are disk prostheses in the Netherlands), it might not be economically worthwhile to advise disk prosthesis. A thorough economic evaluation is warranted.

We will not advocate new studies including new patients. One possibility would be to collect all individual patient data in the numerous randomized controlled trials that have been performed comparing ACDA with ACDF by fixation with plate by an independent researcher who has no relation to the industry and does not favour one method above another. Focus should be on the difference in proportion of good outcome. Whether disk prosthesis should be advised so as to prevent adjacent segment disease cannot be concluded based on the results of this study. Cost analysis in relation to number to treat is important.

### Limitations

Ending the trial before reaching the calculated sample size might be explained as a major flaw. As explained in the Methods section, we could not justify continuation of inclusion. We felt a major obligation to follow the patients and report on the results. Given the presented results following the protocol, in our opinion, the conclusion would be the same as when the calculated sample size was achieved. Because of these sample size constraints, the risk of not detecting even modest changes is still present, but nevertheless we would like to describe the findings as inconclusive.[30] However, recent insights about the definition of a good outcome[20] make us doubtful of the correctness of this decision. Comparing the proportions of good outcome, extending the trial could probably have led to a more conclusive statement about a difference in treatments.

The long time to include patients might also be addressed as a flaw. However, since the start of the trial, operative techniques have not been changed, and this will not have influenced the results. Although the trial was developed initially as a multi-center trial, other initially supporting centers did not participate. The mono-center execution of the trial might be defined as a flaw as well, but in our opinion, this will not affect generalizability. The inclusion criteria were very clear, the operative method is not exclusive, the decision to offer new surgery was also clearly described, and the primary outcome NDI is a patient-reported outcome that has been validated worldwide.

We think that this small deviation of the original protocol by adapting the maximum age did not influence the results since movement of the involved disc level at the dynamic X Rays was required for inclusion.

Not reporting the short-term outcomes at 3 months and 1 year did not influence the interpretation of the results, since after 6 weeks postoperatively the results did not alter. This is in accordance with our experience in daily clinical practice. Major improvements in the clinical situation are not expected anymore after the first postoperative out patient clinical visit (approximately six weeks postoperatively).

Another shortcoming is the decision to make the patient responsible for completing the questionnaires without strict control. Therefore, the response gradually decreased during follow-up. However, at the last follow-up, the response rate was nearly 100%, probably because of a more active attitude from the researchers. This contributed to a good response regarding primary outcome NDI, NRS, and reoperations. We are aware that a VAS measured in millimetres is not similar to the NRS in scale 0 to 10, but comparison of severity of pain is still possible [19]. Therefore, the VAS as part of the secondary outcome MPQ-DLV can be compared with the NRS arm and neck. These outcomes are relevant to patients. The effect of blinding is always a subject of discussion. However, since patients always see their postoperative radiograph, blinding was not possible. Since we advocated the trial to the patients by emphasizing that we really did not know what the best treatment was, the effect of not blinding will be minimal. Further, one might criticize the decision to analyse the whole group including those patients with additional surgery after the index surgery. In our opinion, though, this resembles the daily clinical practice, since patients are interested in the final result after a certain treatment including additional operative or non-operative treatments. This might result in worse treatment results because of complaints occurring because of a second operated level. Therefore, it could be considered as a pragmatic solution, since we do not optimize our analysis only in order to determine efficacy.[17]

Selection bias based on failure for concealment might have occurred. However, since the surgeons did not have any preference for a method, the patients were included after they had the possibility to consider participation, and the allocation was known the evening before surgery we think the change for this kind of bias is minimized. In fact, none of the patients refused the allocated type surgery, nor did the surgeons refuse to perform it.

## Conclusion

This randomized trial could not detect a difference between three surgical modalities for treating a single-level degenerative disk disease. Anterior cervical discectomy without implant seems to be similar to anterior cervical discectomy with fusion by cage stand-alone or with disk prosthesis. Due to the small study sample size, this statement should be considered as inconclusive so far. Although a difference was noted in the incidence of adjacent disc disease a definitive conclusion can not be made due to the small sample size.

## Supporting information

S1 File Consort-2010-checklist. (DOC)

S2 File Protocol cervicale discectomie. (DOCX)

S3 File Protocol English anterior cervical discectomy. (DOCX)

S4 File Individual patient data. (SAV)

S5 File NDI at start and follow-up. (SAV)
