# Supplementary material for: What’s the best surgical treatment for patients with cervical radiculopathy due to single-level degenerative disease? A randomized controlled trial
Source: PLoS One. 2017 Aug 29;12(8):e0183603. doi: 10.1371/journal.pone.0183603 (PMC5574537; doi:10.1371/journal.pone.0183603)
Supplement: S2 File — (DOCX) [file pone.0183603.s002.docx]

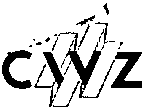


Cervicale anterieure discectomie: zonder fusie, met fusie middels plaatsing van cage of met plaatsing Bryan's cervicale discusprothese?:

een prospectief gerandomiseerde, niet geblindeerde studie.

2 0 0 2 *I* 2 0 0

Hoofdonderzoeker:

R. Donk

Afdeling Orthopedie

Canisius Wilhelmina Ziekenhuis Weg door Jonkerbos 100 Nijmegen

In samenwerking met:

R.H.M.A. Bartels

Neurochirurgisch Centrum Nijmegen Universitair Medisch Centrum St. Radboud

R. Postlaan 4 Nijmegen

N

"*;*-*;t*'


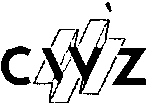


**Inleiding**

De cervicale anterieure discectomie is een internationaal erkende en inmiddels wijd verbreide operatieve behandeling van cervicale hernia's en osteophyten. De manier waarop de operatie wordt uitgevoerd is nagenoeg standaard. Verschillen in het verwijderen van de tussenwervelschijf zijn nauwelijks aanwezig. Een hernia nuclei pulposi, osteophyten of verkalkt ligamentum longitudinale posterior dei compressie geven, moeten worden verwijderd waardoor neurale structuren weer geheel vrij komen te liggen

Verschillende ideeën bestaan ten aanzien van het vervolg van de operatieve behandeling. Twee denkwijzen bestaan: enerzijds zou discectomie alleen voldoende zijn,

anderzijds wordt het nastreven van een fusie sterk bepleit. Sedert korte tijd is er nog een derde

**4i1** mogelijkheid bijgekomen waar nog geen literatuur over bestaat: de Bryan's discusprothese.

De voor- en nadelen van de verschillende benaderingen zullen achtereenvolgens worden besproken.

Vooropgesteld dient te worden dat alle drie de operatiemethoden gelijk zijn vanaf de huidincisie tot het moment dat de discectomie heeft plaatsgevonden en de comprimerende factoren zijn weggenomen. Vindt geen fusie of implantatie van een discusprothese plaats, wordt de wond gesloten. Anders volgt de implantatie van het fusiemateriaal of de discusprothese, waarna tot wondsluiting wordt overgegegaan.

De theoretische voordelen van cervicale discectomie met fusie zijn: herstel van de normale cervicale curvatuur, distractie van de foramina en het voorkomen van kyphosering

2 17 20

van de cervicale wervelkolom. 1; ; ;

- Fusie kan op verschillende manieren worden

bewerkstelligd. De meest gebruikte methode is interpositie van een botspaan (graft) uit de crista. Nadelen van deze benadering zijn de mogelijke complicaties van de donor site (tot 20%

29

beschreven) S;lS;

: meralgia paresthetica, infectie, bekkenfractuur, chronische pijn kurmen

volgen na de initiële pijn, zwelling en hematoom. Dit kan ondervangen worden door gebruik te maken van een allograft. Inzakken van de botspaan (zowel allo- als autograft) en extrusie van de spaan zijn andere problemen die kurmen optreden. Het inzakken gebeurt niet als gebruik wordt gemaakt van een cage die is gemaakt van titanium, carbon fibre of PEAK. In eerste instantie zorgt de cage ervoor dat de hoogte van de tussenwervelschijf behouden blijft,

terwijl het bot door de cage heen kan groeien totdat uiteindelijk fusie is verkregen 6;

22

- De cage

zelf zal niet inzakken, maar kan wel inzakken in een wervellichaam. Het uitbreken van een spaan kan voorkomen worden door interne fixatie in de vorm van een plaat. Ook het verlies

u

N

0


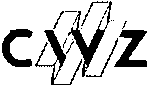


aan lordose wordt met plaat beter bestreden en het inzakken van de graft treedt minder

26

frequent op I O;l 6;

. Door gebruik te maken van een cage gevuld met een botvervangend

materiaal worden alle boven beschreven problemen met uitzondering van het inzakken van de

28 30

cage in een wervel 13; ; .

De voordelen van een discectomie zonder fusie zijn de afwezigheid van de complicaties die gepaard gaan met het nemen van de botspaan 19• De operatie duurt ook korter23 . Echter bij het merendeel van de patiënten treedt ook een fusie op, terwijl deze niet wordt nagestreefd. Veelal is dit in een niet optimale, kyphotische stand tengevolge van het

inzakken van de tussenwervelruimte. Het verlies aan hoogte kan weer aanleiding geven tot verkleining van de neuroforamina met wortelcompressie tot gevolg.

Al dan niet beoogde fusie leidt tot verhoogde belasting van de belendende niveaux.

e

Ook de bewegingen in de aanpalende disci neemt toe. Vervroegde degeneratie (adjacent disc disease) is het gevolg. Adjacent disc disease heeft een jaarlijkse incidentie van 2.5% per jaar

21

4;l4;

. Operatie is vaak noodzakelijk.

Een probleem dat met name bij beoogde fusie ter sprake komt is de niet optredende

verbening (pseudoarthrose ). Het spreekt voor zich dat pseudoarthrose ook optreedt bij de discectomie. zonder de beoogde fusie 1• Alleen indien fusie nagestreefd wordt dient daadwerkelijk ook ingroei vanuit de wervels in de graft plaats te vinden. De incidentie

2 25 27 30

varieert van 3 tot 50 %7;I0; o;

; ; . De incidentie is laag als slechts een niveau behandeld

wordt en neemt toe naarmate meerdere niveaux geopereerd worden. Gebruik van een interne fixatie vermindert de kans op pseudoarthrose 16• Het is echter niet gezegd dat een pseudoarthrose automatisch een slechter klinisch resultaat inhoudt. Klachten bestaan veelal uit

•

nekpijn die afhankelijk is van mechanische belasting. Soms treden de klachten jaren na een

20 25 30 8

operatie pas op

; ; . Reoperatie behoort tot de mogelijkheden .

Tot dusver is het niet duidelijk of cervicale anterieure discectomie met fusie beter is dan zonder fusie. Vergelijkende studies laten op lange termijn geen verschil zien tussen cervicale discectomie met of zonder fusie. Sommige studies tonen aan dat de mensen met

24

fusie minder nekpijn en intrascapulaire pijn te hebben en eerder het werk te hervatten 3; ,

andere beweren juist het tegendeel 9. Aan complicaties van het nemen en plaatsen van een

graft staan zij die geen fusie ondergaan natuurlijk niet bloot. Ondanks de vele studies is klasse 1 bewijs voor de superioriteit of inferioriteit van een van beide methoden nog nooit geleverd.

Sinds kort bestaat een derde mogelijkheid: de Bryan's cervicale discusprothese. De discusprothese kan als een kunstgewricht worden beschouwd met een beweeglijkheid binnen


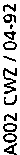


cq/;z.

fysiologische grenzen. Idealiter herstelt een operatie de normale vorm en ook de beweeglijkheid van de wervelkolom nadat de comprimerende factoren weggenomen zijn. Door implantatie van een Bryan's cervicale discusprothese wordt dit nagestreefd. Na een standaard cervicale discectomie wordt de discusprothese geïmplanteerd. Door de beweeglijkheid van de prothese is de beweeglijkheid van het geopereerde niveau gelijk of wellicht beter dan preoperatief. Daarnaast blijft de hoogte van de tussenwervelruimte bewaard en daarmee de hoogte van de neuroforamina. In een aantal selecte centra in Europa is tot op heden ervaring hiermee opgedaan. De langste follow-up bedraagt tweeëenhalf jaar.

Complicaties gerelateerd aan de prothese hebben zich niet voorgedaan !!;!2. De Bryan's cervicale discusprothese heeft CE markering, is vrij verkrijgbaar en mag geïmplanteerd *{* worden buiten onderzoeksprotocollen.

Er bestaat nog steeds geen consensus over de vraag welke methode de beste is voor de operatieve behandeling van een cervicale radiculopathie door een HNP of osteophyt: cervicale discectomie met of zonder fusie. De komst van de cervicale discusprothese is aanleiding om een vergelijkend onderzoek tussen de verschillende methoden te starten.

**Doelstelling**

•

Middels een studie vergelijken of er een voordeel is van een van de drie methoden boven de andere: cervicale discectomie zonder aanvullende maatregelen (1), met fusie gebruik makend van cage en botvervangend materiaal (2) en tot slot met implanatie van Bryan's cervicale discusprothese (3). Voor fusie met cage en botvervangend materiaal is gekozen vanwege aanwezige ervaring van de eventuele operateurs enerzijds, en doordat door de afwezigheid van de complicaties samenhangend met een graft een betere vergelijking met de andere methoden mogelijk is. Omdat herstel van neurologische uitval of vermindering van radiculaire pijnklachten gerelateerd is aan de discectomie sec en niet aan het al dan niet uitvoeren van een fusie, zal de beoordeling hiervan wel worden meegenomen in het onderzoek, maar geen criterium vormen om de verschillende methoden te vergelijken.

Daarentegen verdient vermindering van pijnklachten in de nek specifieke aandacht alsook het optreden van adjacent disc disease.


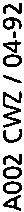


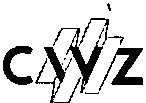


**Hypothesen:**

- - 1. Ho: Nekklachten zijn aanzienlijk minder na cervicale anterieure discectomie na implantatie van een discusprothese, dan na discectomie sec of met implantatie van een cage.

1. H0 : Na implantatie van een cervicale discusprothese gaan patiënten eerder aan het werk dan diegene die geen discusprothese hebben.
2. H0 : Na 5 jaar is er minder uitgesproken radiologisch aantoonbare degeneratie van belendende disci na het uitvoeren van een cervicale discectomie met implantatie van een discusprothese dan na discectomie sec of na discectomie met implantatie van een cage.

### •


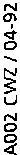


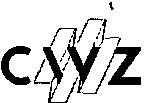


**Methoden:**

De opzet is een prospectief gerandomiseerde, niet geblindeerde studie, die zo wordt opgezet dat uitbreiding naar meerdere centra (multicenter - studie) mogelijk is.

Drie patiëntengroepen worden vergeleken:

- 1. cervicale anterieure discectomie zonder fusie of implantaat,
  2. cervicale anterieure discectomie met fusie middels cage en botvervangend materiaal
  3. cervicale anterieure discectomie met Bryan's cervicale discusprothese.

Patiënten zullen gerandomiseerd worden aan de hand van enveloppen, die bekend zijn bij een onafhankelijke instantie (secretariaat orthopedie CWZ). Randomisatie vindt alleen plaats

**e** nadat voldaan is aan inclusie - en exclusiecriteria (zie Tabel 1) en informed consent is

verkregen. Informed consent wordt verkregen nadat de patiënt 14 dagen bedenktijd heeft gehad en hij/zij gelegenheid heeft gehad de aangeboden informatie (mondeling/schriftelijk) te overdenken. Na inclusie zal een onafhankelijk neuroloog patiënt onderzoeken. Dit geschiedt op de afdeling op de dag voor de operatie. Preoperatief dient het volgende radiologische onderzoek aanwezig te zijn: MRI, X - CWK AP/Lateraal met flexie/deflexie - opnamen en voor diegenen die gerandomiseerd zijn voor implantatie van een discusprothese een CT van de wervelkolom (betroffen discusniveau). Tevens zullen de patiënten gevraagd worden de volgende score lijsten in te vullen: SF-36, MDQ-DLV en neck disability index. Daarnaast wordt middels anamnese het rookgedrag vastgelegd, alsook de aanwezigheid van fibromyalgie, ME, en whiplash. De invloed van de afzonderlijke factoren op het pijngedrag zal worden beoordeeld.

Vervolgens worden de patiënten ingepland voor operatie. De operateurs dienen bedreven te zijn in elk van eerder genoemde technieken, opdat de mogelijkheid van verschil in methoden door andere techniek van operateurs wordt uitgebannen . Blijkt tijdens een operatie geen discusprothese geplaatst te kunnen worden, zal eerst een fusie middels cage plaatsing worden nagestreefd, waardoor patiënt in groep 2 terechtkomt (intention-to-treat). Lukt dit ook niet volgt discectomie zonder aanvullende maatregelen. Na de operatie worden patiënten zonder externe hulpmiddelen aangespoord zo snel mogelijk weer hun oude activiteiten op te pakken. In de direct postoperatieve fase wordt het gebruik van pijnstilling bijgehouden. Tevens wordt de lengte van het verblijf in het ziekenhuis geregistreerd. Een controle X-CWK AP/Lateraal wordt 1 dag na operatie gemaakt.


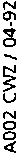


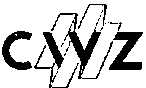


Poliklinische controles volgen na zes weken, drie maanden en een jaar. Nadien vindt jaarlijkse controle plaats. In het eerste jaar vindt naast controle controle bij de operateur controle door een onafhankelijk neuroloog plaats. De neurologische afwijkingen worden alleen door hem/haar beoordeeld. Nadien vindt alleen nog maar controle bij de operateur plaats. Bij ieder poliklinisch bezoek wordt patiënt verzocht de eerdergenoemde scorelijsten in te vullen. Totdat volledige werkhervatting heeft plaatsgevonden, houdt patiënt een dagboek waarin hij/zij aangeeft wat de beperkingen zijn en wat het eventuele pijnmedicatie gebruik is.

Radiologisch onderzoek vindt eveneens bij elke controle plaats. Iedere keer wordt een X-CWK *API* lateraal met flexie/deflexie gemaakt. Vorm van de wervelkolom wordt gescoord, mate van fusie en beweeglijkheid van het geopereerde niveau. Dit geschiedt door

een onafhankelijk radioloog, die ervaren is in de beoordeling van wervelkolompathologie.

**e** Een jaar na de operatie wordt een CT gemaakt van het geopereerde niveau. De mate van bot

doorgroei wordt beoordeeld en in geval van de discusprothese wordt gekeken er aanwijzingen zijn voor onvoldoende inbouw in de aangrenzende wervels (lucentie). Vijf jaar na operatie wordt een MRI gemaakt om de disci van de aanpalende niveaux te beoordelen. Tevens wordt het aantal heroperaties aan de halswervelkolom binnen de afzonderlijke groepen gescoord.

Eindpunten worden bereikt voor hypothese 1 na 1 jaar en voor hypothese 2 na 5 jaar.

| INCLUSIE | EXCLUSIE |
| --- | --- |
|  |  |
| Leeftijd: 18 - 50 jaar | Myelopathie |
| Radiculopathie | Eerdere operatie aan cervicale wervelkolom |
| Monosegmentaal | Psychiatrische voorgeschiedenis |
| Osteophyt/HNP | Verwikkeld in aansprakelijkheidsprocedure |
| Beweeglijkheid niveau op X-CWK | Alcoholisme |
|  | Levensverwachting < 1 jaar |
|  | Zwakzinnigheid |
|  | Nederlandse taal niet machtig |

### •

Tabel 1: in - en exclusie criteria voor randomisatie

**Berekening sample** sizes:

N

"g'

N

0


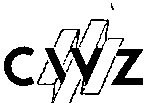


Op basis van de beschikbare literatuur kunnen we veronderstellen dat zowel voor de cervicale discectomie zonder als met fusie een excellent resultaat ten aanzien van de nekklachten in ongeveer 60 % van de gevallen bereikt wordt. De verwachting is dat dit percentage bij een discusprothese hoger ligt (80%). Het primaire eindpunt is na 1 jaar waarbij gekeken wordt naar de proportie geheel klachtenvrije patiënten bij de afzonderlijke groepen.

Indien een D = 0,05 en een power van 80% wordt aangenomen, zijn 81 patiënten per groep nodig om een verschil aan te tonen bij een tweezijdige toetsing.

**Belasting voor patiënten:**

Preoperatief gebeuren geen andere zaken dan normaal met uitzondering van het invullen van de vragenlijsten en het onderzoek door de neuroloog. Dit laatste legt geen beslag op de tijd van de patiënt, omdat dit klinisch gebeurt. Het invullen van de vragenlijsten vraagt twintig minuten.

•

Postoperatief vinden de controles tot 1 jaar plaats zoals deze nu ook plaatsvinden. Ook nu is de controle door de neuroloog extra. Dit vergt 10 à 15 minuten per keer en vindt plaats aansluitend aan de reguliere poliklinische controles. Wederom moeten de vragenlijsten ingevuld worden. De radiologische onderzoeken gedurende het eerste jaar zijn standaard. De CT scan wordt momenteel regelmatig gemaakt om fusie te beoordelen, maar niet standaard.

Dit zou als extra omschreven moeten worden. Dit leidt wel tot een eenmalige geringe stralenbelasting en een tijdsinvestering van ongeveer een uur. Er wordt na gestreefd om dit samen te laten vallen met de datum van een poliklinisch bezoek opdat het verlies van tijd door reizen geminimaliseerd wordt.


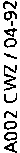


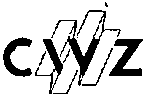


De jaarlijkse controles na 1jaar postoperatief moeten als extra omschreven worden.

De tijdsinvestering bestaat uit reistijd, polikliniek bezoek en invullen van vragenlijsten. Radiologisch 011derzoek vindt direct voor het poliklinische bezoek plaats. Radiologische belasting qua straling is vrijwel nihil. De MRI die na vijf jaar gemaakt wordt levert in het geheel geen stralenbelasting op.

### •


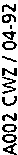


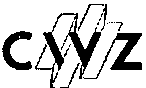


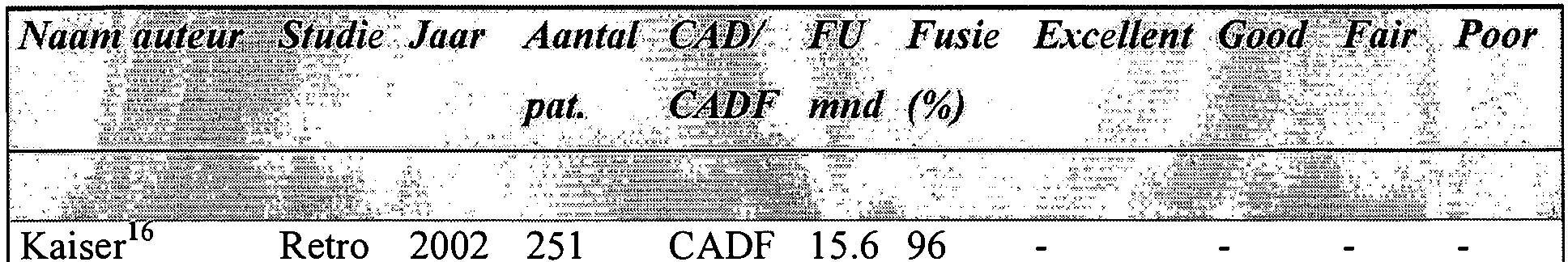


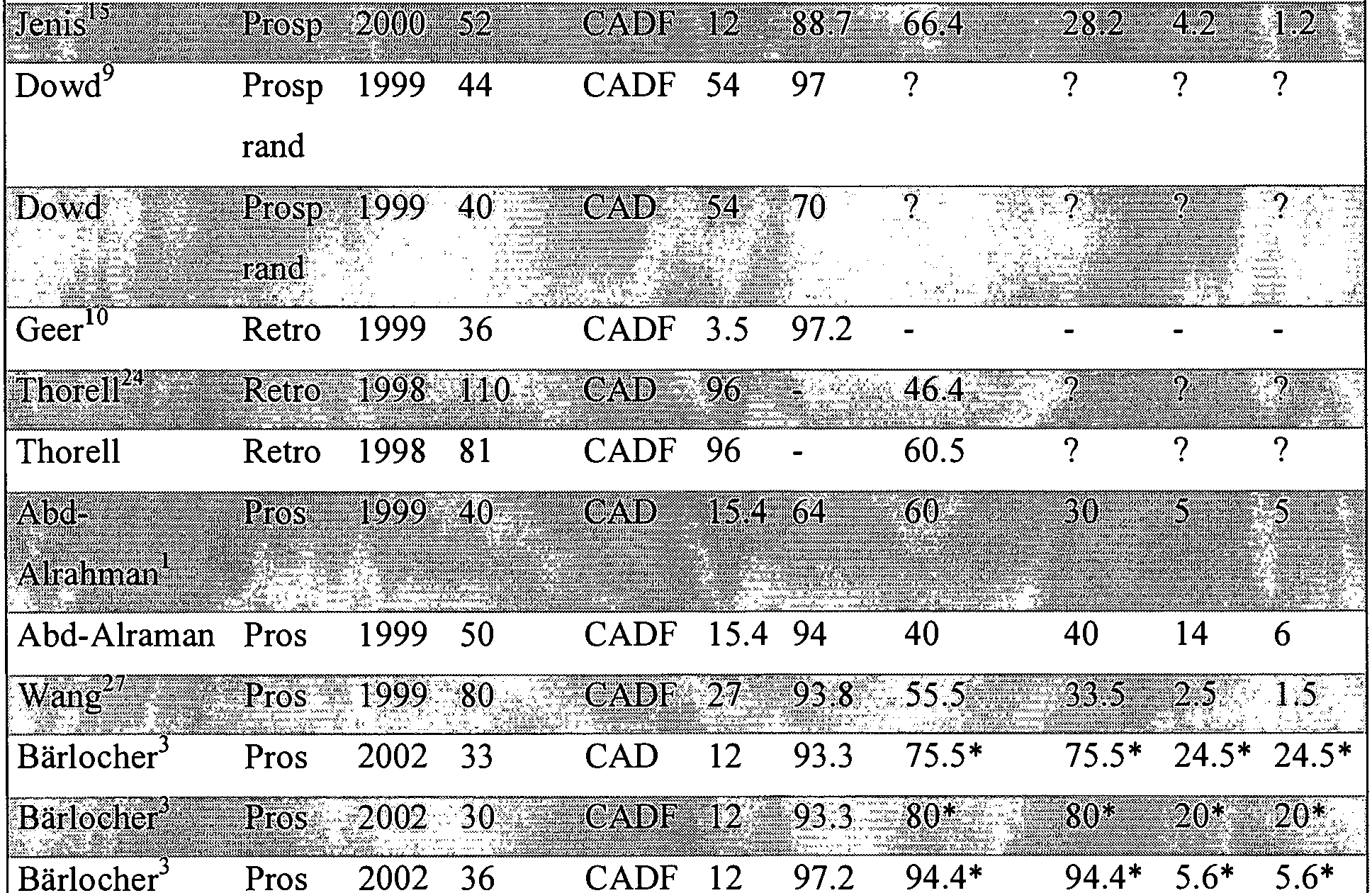


Cage

* *excellentlgood enfair/poor samen gevoegd*

N

"-'

u

Ng

<!

Overzicht resultaten studies


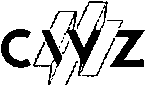


References

1. Abd-Alrahman N, Dokmak AS, Abou-Madawi A: Anterior cervical discectomy (ACD) versus anterior cervical fusion (ACF), clinical and radiological outcome study. **Acta Neurochir.(Wien)** 141:1089-1092, 1999
2. Bartels RH, Donk R, van-Azn RD: Height of cervical foramina after anterior discectomy and implantation of a carbon fiber cage. **J.Neurosurg.** 95:40-42, 2001
3. Bärlocher CB, Barth A, Krauss JK, et al: Comparative evaluation of microdiscectomy only, autograft fusion, polymethylacrylate interposition, and threaded titanium cage fusion for treatment of singel-level cervical disc disease: a prospective randomized study in 125 patients. **Neurosurg.Focus** 12:1-7, 2002
4. Benzel EC: Spinal fusion, in Benzel EC (ed): **Biomechanics of spine stabilization.**

. Rolling Meadows, American Association of Neurological Surgeons:l 21-134, 2001

1. Bishop RC, Moore KA, Hadley MN: Anterior cervical interbody fusion using autogeneic and allogeneic bone graft substrate: a prospective comparative analysis. **Journal of Neurosurgery** 85:206-210, 1996
2. Brooke NS, Rorke AW, King AT, et al: Preliminary experience of carbon fibre cage prostheses for treatment of cervical spine disorders. **Br.J.Neurosurg.** 11:221- 227, 1997
3. Casey AT: Bone grafts and anterior cervical discectomy--lack of evidence, but no lack of opinion. **Br.J.Neurosurg.** 13:445-448, 1999

N

-

q>

;g

u

N

0

0

<!


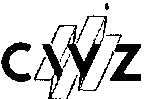


1. Coric D, Branch C-LJ, Jenkins JD: Revision of anterior cervical pseudoarthrosis with anterior allograft fusion and plating. **J.Neurosurg.** 86:969-974, 1997
2. Dowd GC, Wirth FP: Anterior cervical discectomy: is fusion necessary? **J.Neurosurg.**

90:8-12, 1999

1. Geer CP, Papadopoulos SM: The argument for single-level anterior cervical discectomy and fusion with anterior plate fixation. **Clin.Neurosurg.** 45:25-29, 1999
2. Goffin J, Casey A, Kehr P, et al: Preliminary clinical experience with the Bryan cervical disc prosthesis. **Neurosurgery** 51:840-847, 2002
3. Goffin, J., Casey, A., Kehr, P., Liebig, K., Lind, B., Logroscino, C., Pointillart, V., Van Calenbergh, F., and Van Loon, J. Intermediate follow-up after treatment of degenerative disc disease with Bryan™ vervical disc prosthesis. **l**8th annual meeting(Multidisciplinary approaches to the cervical spine. Cervical Spine Research Society. Paris, France). 2002. (GENERIC)

Ref Type: Conference Proceeding

1. Hacker RJ, Cauthen JC, Gilbert TJ, et al: A prospective randomized multicenter clinical evaluation of an anterior cervical fusion cage. **Spine** 25:2646-2654, 2000
2. Hilibrand AS, Yoo JU, Carlson GD, et al: The success of anterior arthrodesis adjacent to a previous fusion. **Spine** 22:1574-1579, 1997
3. Jenis LG, An HS, Simpson JM: A prospective comparison of the standard and reverse robinson cervical grafting techniques: radiographic and clinical analyses. **J.Spinal.Disord.** 13:369-373, 2000


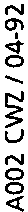


.

*r:t}-,*

***CL}V) Z***

1. Kaiser MG, Haid RWJ, Subach BR, et al: Anterior cervical plating enhances arthrodesis after discectomy and fusion with cortical allograft. **Neurosurgery** 50:229-236, 2002
2. Laing RJ, Ng I, Seeley HM, et al: Prospective study of clinical and radiological outcome after anterior cervical discectomy. **Br.J.Neurosurg.** 15:319-323, 2001
3. Martin G-JJ, Haid R-WJ, MacMillan M, et al: Anterior cervical discectomy with freeze- dried fibula allograft. Overview of 317 cases and literature review. **Spine** 24:852-858, 1999
4. Maurice- Williams RS, Dorward NL: Extended anterior cervical discectomy without fusion: a simple and sufficient operation for most cases of cervical degenerative disease. **Br.J.Neurosurg.** 10:261-266, 1996
5. Phillips FM, Carlson G, Emery SE, et al: Anterior cervical pseudarthrosis. Natural history and treatment. **Spine** 22: 1585-1589, 1997
6. Pointillart V, Carlier Y, Pedram M, et al: Outcome of cervical spinal levels adjacent to anterior fusion: long term follow up. **Eur.Spine J. 11 (suppl** l):s72002(Abstract)
7. Salame K, Ouaknine GER, Razon N, et al: The use of carbon fibre cages in anterior cervical interbody fusion. Report of 100 cases. **Neurosurg.Focus 12: 1 -4,** 2002
8. Savitz MH: Anterior cervical discectomy without fusion or instrumentation: 25 years' experience. **Mt.Sinai.J.Med.** 67:314-317, 2000


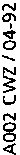


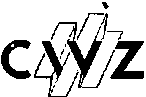


1. Thorell W, Cooper J, Hellbusch L, et al: The long-term clinical outcome of patients undergoing anterior cervical discectomy with and without intervertebral bone graft placement. **Neurosurgery** 43:268-273, 1998
2. Tribus CB, Corteen DP, Zdeblick TA: The efficacy of anterior cervical plating in the management of symptomatic pseudoarthrosis of the cervical spine. **Spine**

**24:** 860-864, 1999

1. Troyanovich SJ, Stroink AR, Kattner KA, et al: Does anterior plating maintain cervical lordosis versus conventional fusion techniques? A retrospective analysis of patients receiving single-level fusions. **J.Spinal.Disord.Tech.** 15:69-74, 2002
2. Wang JC, McDonough PW, Endow K, et al: The effect of cervical plating on single- level anterior cervical discectomy and fusion. **J.Spinal.Disord.** 12:467-471,

. 1999

1. Whitecloud TS: Modem altematives and techniques for one-level discectomy and fusion. **Clin.Orthop.** 67-76, 1999
2. Wigfield CC, Nelson RJ: Nonautologous interbody fusion materials in cervical spine surgery: how strong is the evidence to justify their use? **Spine** 26:687-694, 2001
3. Zdeblick TA, Hughes SS, Riew KD, et al: Failed anterior cervical discectomy and arthrodesis. Analysis and treatment of thirty-five patients. **J.Bone Joint Surg** Am. 79:523-532, 1997
