# Supplementary material for: What’s the best surgical treatment for patients with cervical radiculopathy due to single-level degenerative disease? A randomized controlled trial
Source: PLoS One. 2017 Aug 29;12(8):e0183603. doi: 10.1371/journal.pone.0183603 (PMC5574537; doi:10.1371/journal.pone.0183603)
Supplement: S3 File — (DOCX) [file pone.0183603.s003.docx]

Anterior cervical discectomy: without fusion, with fusion using cage placement or placement of Bryan’s cervical disc prosthesis?:

A prospective, randomized, non-blinded study.

Primary Investigator:

Roland Donk

Afdeling Orthopedie

Canisius Wilhelmina Ziekenhuis Weg door Jonkerbos 100 Nijmegen

In collaboration with:

Ronald Bartels

Neurochirurgisch Centrum Nijmegen Universitair Medisch Centrum St. Radboud

R. Postlaan 4

Nijmegen

**Introduction**

The anterior cervical discectomy is an internationally recognized and now widespread surgical treatment of cervical herniated discs and osteophytes. The way in which the operation is carried out is virtually standard. Differences in the removal of the intervertebral disc are hardly present. A herniated nucleus pulposus, osteophytes or calcified posterior longitudinal ligament with compression should be removed in order to decompress neural structures completely.

Several ideas exist with regard to the remainder of the operative treatment. Two schools of thought exist: according to one only discectomy would be enough, the other pursues fusion. Recently a third method is possible, of which no literature exists: Bryan's disc prosthesis. The advantages and disadvantages of different approaches will be discussed subsequently.

It should first be noted that all three methods of operation are the same from the skin incision until the moment that the discectomy has been taken place and the compressing factors have been removed. If fusion or the implantation of disc prosthesis is not pursued, the wound is closed. Otherwise, the implantation of fusion material or the disc prosthesis will follow the discectomy, and the wound is closed.

The theoretical benefits of cervical discectomy with fusion are: restoration of normal cervical curvature, distraction of the foramina and prevention of kyphosis of the cervical spine. Fusion can be achieved in various ways. The most common method is interposition of a bone graft taken from the iliac crest. Disadvantages of this approach are the potential complications to the donor site (up to 20%): meralgia paresthetica, infection, pelvic fracture, chronic pain can occur after the initial pain, swelling, and hematoma. Making use of an allograft can prevent these. Collapse of the graft (both allogeneic as autograft) and extrusion of the graft are other problems that occasionally occur. A cage made of titanium, carbon fiber or PEAK will not collapse. In first instance, the cage ensures that the height of the intervertebral disc is retained while the bone can grow through the cage until fusion eventually is obtained. The cage itself will not collapse, but it can subside into a vertebral body. Luxation of the graft can be prevented by internal fixation using a plate. A plate better prevents the loss of lordosis and the collapse of the graft occurs less frequent. A cage filled with a bone substitute material prevents all the problems described above with the exception of the collapse of the cage in a vertebral body.

The advantages of a discectomy without fusion are the absence of the complications associated with the bone graft. The operation time is also less. However, in the majority of patients fusion will take place, while it is not pursued. In many cases, this is in a non-optimal, kyphotic position due to the collapse of the intervertebral space. The loss of height may again give rise to reduction of the foramina with root compression as a result.

Whether or not pursued fusion, it leads to increased stress on the adjacent levels. The movements in the adjacent intervertebral discs increase. Early degeneration (adjacent disc disease) is the result. Adjacent disc disease has an annual incidence of 2.5% per year. Operation is often necessary.

The absence of fusion whereas it was intended (nonunion) might be a problem. It is obvious that a nonunion might also occur if only a discectomy was performed. Actual ingrowth should occur from the vertebrae in the graft if fusion was expected. The incidence ranges from 3 to 50%. The incidence is low when only one level is treated and increases, as more levels were included in surgery. Use of an internal fixation reduces the risk of nonunion. It is not said that a nonunion automatically implies a worse clinical outcome. Complaints often include neck pain depending upon mechanical load. Occasionally, the symptoms occur years after surgery.

So far it is not clear whether anterior cervical discectomy with fusion is better than without. Comparative studies on long-term do not show any difference between cervical discectomy with or without fusion. Some studies show that people with fusion tend to have less neck pain and intrascapular pain and earlier return to work. Others just argue the opposite. Those who do not undergo fusion of course are not exposed at complications of taking and placing a graft. Despite the many studies, class 1 evidence showing the superiority or inferiority of one of the two methods has never delivered.

Recently, there is a third possibility: Bryan's cervical disc prosthesis. The disc prosthesis can be regarded as an artificial joint with mobility within physiological limits. Ideally, an operation recovers its normal shape, and also the mobility of the spine after the compressing factors have been removed. This is pursued by the implantation of a Bryan's cervical disc prosthesis. After a standard cervical discectomy the disc prosthesis is implanted. Because of the mobility of the prosthesis is the mobility of the operated level equal or possibly better than preoperatively. In addition, the height of the intervertebral space is maintained, and thus the height of the foramina. In a number of select centers in Europe has hitherto been gained experience. The longest follow-up is two and a half years. Complications related to the prosthesis have not occurred. Bryan's cervical disc prosthesis has CE marking, is freely available and can be implanted outside of research protocols.

There is still no consensus as to which method is the best for the surgical treatment of cervical radiculopathy by herniated intervertebral disc or osteophyte: cervical discectomy with or without fusion. The current availability of the cervical disc prosthesis is reason to initiate a comparative study between the different methods.

**Objective**

Comparing whether an advantage of one of the three methods exists above the other: cervical discectomy without additional measures (1), with fusion using cage and bone substitute material (2) and finally with implantation of Bryan's cervical disc prosthesis (3). The choice for fusion by cage with bone-replacement material is made 1) because of existing experience of the surgeons, and 2) due to the absence of the complications associated with a graft a better comparison with the other methods is possible. Because recovery of neurological deficit or reduction of radicular pain is related to the discectomy only and whether a fusion procedure is performed, the assessment hereof will done, but do not constitute a criterion for comparing different methods.

By contrast, reduction of neck pain as well as the occurrence of adjacent disc disease will receive attention.

**Hypotheses:**

1) Neck problems are substantially less after anterior cervical discectomy after implantation of intervertebral disc prosthesis, compared to discectomy alone or with implantation of a cage.

2) After implantation of a cervical disc prosthesis return to work is earlier for those who have a disc prosthesis.

3) After 5 years, there is less pronounced demonstrable radiologically degeneration of adjacent discs after performing a cervical discectomy with implantation of an intervertebral disc prosthesis than after discectomy sec or after discectomy with implantation of a cage.

**Methods:**

A prospective, randomized, non-blinded study is designed in such manner that expansion to multiple centers (multicenter - study) is possible.

Three patients groups are compared:

1) Anterior cervical discectomy without fusion or implant,

2) Anterior cervical discectomy with fusion using cage and bone substitute material

3) Anterior cervical discectomy with Bryan's cervical disc prosthesis.

Patients will be randomized using closed envelopes, which are delivered by an independent instance (secretary orthopedics CWZ). Randomization occurs only

after fulfillment of inclusion - and exclusion criteria (see Table 1) and informed consent is obtained. Informed consent is obtained after the patient has had 14 days to make up his mind and he / she has had the opportunity to reflect on the information (verbally / written). After inclusion an independent neurologist will examine the patient. This is done at the department on the day before surgery. Preoperatively the following radiological investigations are mandatory: MRI, X - CWK AP / Lateral with flexion / extension and a CT of the spine for those randomized to implantation of a disc prosthesis (involved discus level). Also, the patients will be asked to complete the following scorecards: SF-36, MDQ-DLV and neck disability index. In addition, a history of smoking behavior will be recorded as well as the presence of fibromyalgia, whiplash, and ME. The influence of the individual factors on pain behavior will be assessed.

The patients are then scheduled for surgery. The surgeons should be experienced in each of the aforementioned techniques, so that the possibility of difference in methods is eliminated by a more or less uniform surgical technique. If during the surgery the placement of a disc prosthesis appears not feasible, placement of a cage will be the next option (intention-to-treat principle is followed). If this is neither possible discectomy without additional measures will be performed. After surgery, patients are encouraged to pick up their old activities as soon as possible without auxiliaries. In the immediate postoperative phase the use of analgesia will be recorded. It also is recorded during the stay in the hospital. One day postoperatively a control X ray will be made.

Outpatient clinic visits follow after six weeks, three months and one year. Thereafter, it will be annually. In the first year in addition to the visit of the surgeon control a visit to an independent neurologist will take place. Neurological abnormalities are only by him / her evaluated. Afterwards the patient will visit only the surgeon. At each outpatient visit the patient is asked to complete the aforementioned score lists. Until full resumption of work has taken place, keep a patient diary in which he / she indicates what the limitations are and if and which pain medication was used.

At each follow up visit the patient was radiologically examined: lateral X ray AP/Lateral/flexion and extension. Sagittal alignment of the spine is evaluated, the degree of fusion and motility of the operated level. An independent radiologist does this with experience in the evaluation of spine pathology.

One year after the surgery a CT is made of the operated level. The degree of bone growth will be assessed and examined in the case of the disc prosthesis there is evidence of insufficient integration into the adjacent vertebrae (lucency). Five years after surgery, an MRI designed to assess the discs of the adjacent levels. Also, the number of re-operations is the cervical spine scored within the individual groups.

Endpoints are for the first hypothesis after 1 year, 2 after 5 years evaluated.

| Inclusion | Exclusion |
| --- | --- |
|  |  |
| Age: 18-50 years | myelopathy |
| Mono-level | Earlier radiculopathy cervical spine surgery |
| Osteophyt / Herniated intervertebral disc | Psychiatric history |
| Mobility level X-CWK | Entangled in liability proceedings |
|  | Alcoholism |
|  | Life expectancy <1 year |
|  | Mentally retardation |
|  | Dutch language not fluently speaking |

**Calculating sample size**

Based on the available literature, we can assume that both the cervical discectomy without fusion as well as with an excellent result with regard to the neck pain is achieved in about 60% of the cases. It is expected that this percentage with a disc prosthesis is higher (80%) (*interpreted as a difference of 20% on the NDI*). If a D = 0.05, and it enters a power of 80%, 81 patients per group are required to show a difference when a two-sided testing.

Burden on patients:

Preoperatively the examination by the neurologist and the completion of the questionnaires is extra. The examination by the neurologist will be done clinically. Completing the questionnaire requires twenty minutes.

Outpatient clinic visits will be scheduled as usual (when the patient does not participate to this trial) until one year postoperatively. Again, the questionnaires have to be completed. The radiological examinations are standard during the first year. The CT scan is being made regularly to assess fusion, but not by default.

This should be described as an extra. This leads to a one-time low radiation dose and a time investment of about an hour. The aim is to make it coincide with the date of an outpatient visit in order to minimize the loss of time by traveling. The annual audits after 1 year postoperatively should be defined as additional.

The time investment is travel time, outpatient visits and completing the questionnaires. Radiological examinations do precede directly outpatient visit place. Exposure of radiation is negligible.

Results of studies


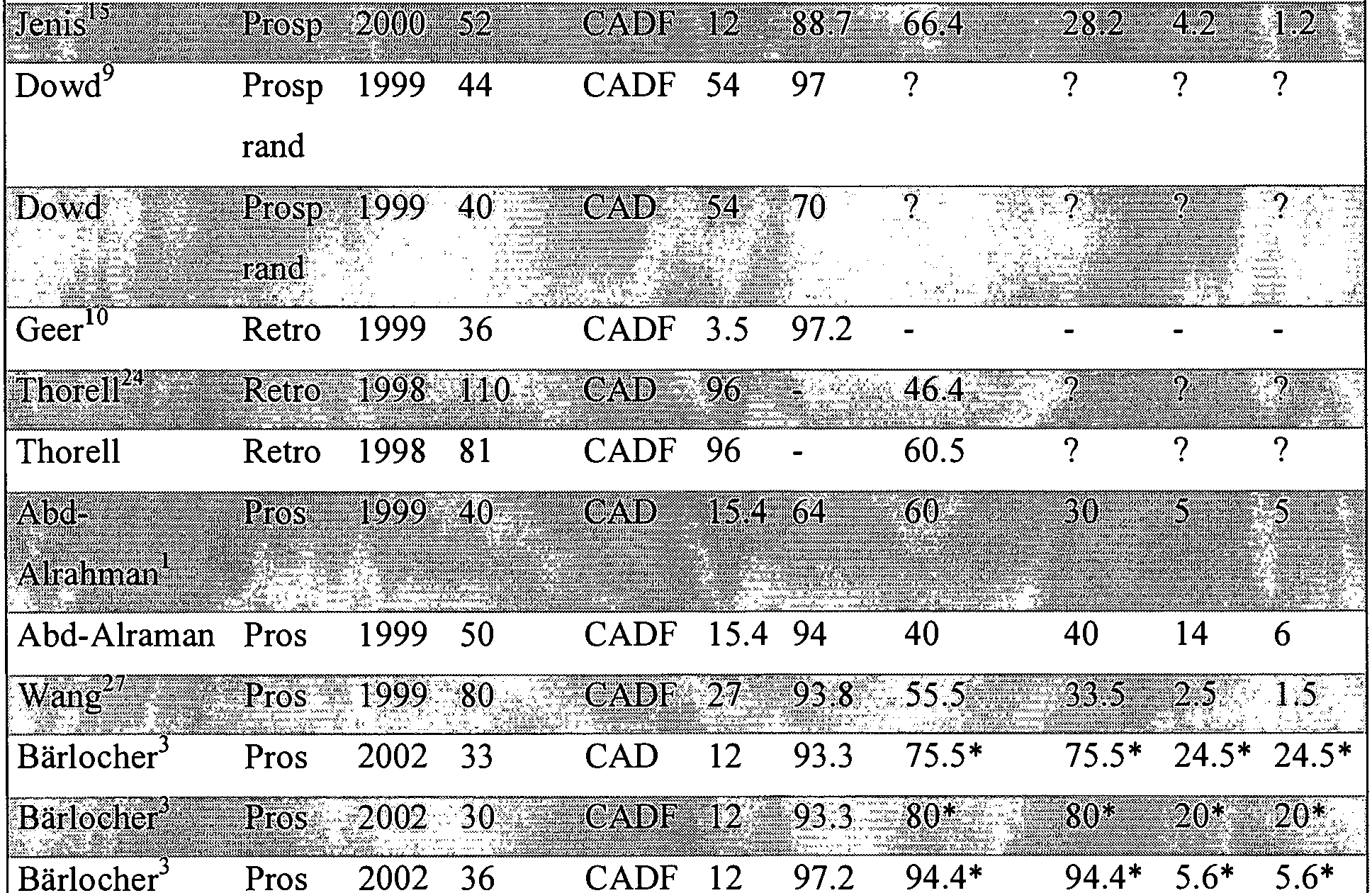


Cage

* *excellentlgood enfair/poor samen gevoegd*


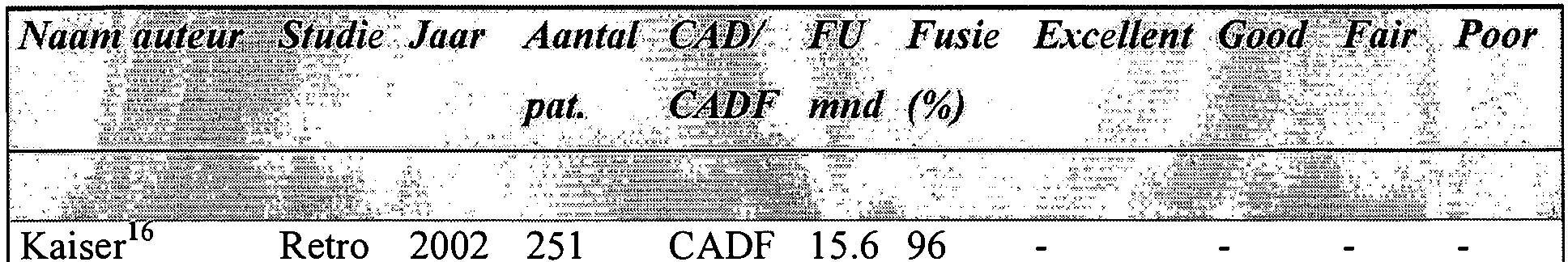


* Dichotomized good/excellent versus fair/poor
